# Associations of Menstrual Cycle Tracking Application Use with Fertility Awareness and Fertility Health Knowledge: A Cross-Sectional Study

**DOI:** 10.3390/healthcare14203425

**Published:** 2026-10-13

**Authors:** Rüveyda Ölmez Yalazı, Selinay Aktaş Demir

**Affiliations:** 1 Department of Midwifery, Faculty of Health Sciences, Fenerbahçe University, 34758 Istanbul, Türkiye; ruveyda.olmez@fbu.edu.tr; 2 Department of Midwifery, Faculty of Health Sciences, Istanbul Atlas University, 34408 Istanbul, Türkiye

**Keywords:** fertility awareness, fertility health knowledge, menstrual cycle, mobile health, women’s health

## Abstract

**Highlights:**

**What are the main findings?**

- Women who used menstrual cycle tracking applications had higher fertility health knowledge and fertility awareness scores than non-users.
- Longer and more frequent application use was positively associated with fertility health knowledge and fertility awareness; symptom-diary users had the highest fertility health knowledge scores.

**What are the implications of the main findings?**

- Menstrual cycle tracking applications may support reproductive health literacy and self-monitoring, but their outputs should be interpreted cautiously because commercial applications vary in scientific validity and algorithmic transparency.
- Women’s health professionals may discuss application use during clinical consultations and, where appropriate, guide women toward tools with transparent, evidence-based methods and clearly stated limitations.

**Abstract:**

**Background/Objectives**: Menstrual cycle tracking applications (MCTAs) are widely used digital health tools that enable women to monitor menstrual characteristics and related symptoms. However, their association with fertility health knowledge and fertility awareness remains insufficiently understood. This study aimed to examine the associations of MCTA use, duration, frequency, and purpose with fertility health knowledge and fertility awareness among women of reproductive age attending a gynecology outpatient clinic. **Methods**: This descriptive, cross-sectional study included 484 women aged 18–49 years who attended the gynecology outpatient clinic of a private hospital in Istanbul, Türkiye, between 1 February and 1 June 2026. Data were collected using a Descriptive Information Form, the Fertility Health Knowledge Scale (FHKS), and the Fertility Awareness Scale (FAS). Descriptive statistics, independent-samples *t*-tests, one-way analysis of variance, Pearson correlation analyses, and simple linear regression analyses were performed. **Results**: MCTA users had significantly higher FHKS total, FAS cognitive awareness, FAS bodily awareness, and FAS total scores than non-users (*p* < 0.001). In the regression models, MCTA use explained 8.5% of the variance in FHKS scores (F = 44.771, R^2^ = 0.085, *p* < 0.001) and 6.6% of the variance in FAS scores (F = 33.884, R^2^ = 0.066, *p* < 0.001). Application-use duration was positively correlated with FHKS (r = 0.171, *p* < 0.001) and FAS (r = 0.144, *p* = 0.001) total scores. Use frequency was also positively correlated with FHKS (r = 0.217, *p* < 0.001) and FAS (r = 0.119, *p* = 0.009) total scores. Women using MCTAs as a symptom diary had the highest FHKS scores (117.35 ± 18.78). Conclusions: MCTA use was associated with higher fertility health knowledge and fertility awareness. MCTAs may be considered as adjunctive self-monitoring tools; however, recommendations should be cautious and limited to applications with transparent, evidence-based methods and clearly communicated limitations.

## 1. Introduction

The menstrual cycle is one of the primary indicators of the healthy functioning of the female reproductive system, the functional integrity of the hypothalamic–pituitary–ovarian axis, and overall physiological status [1,2]. Accurate and regular monitoring of this physiological process, which reflects the general health status of women of reproductive age, plays a critical role in the early detection of potential gynaecological and endocrinological pathologies [3,4]. Furthermore, women’s tracking of their own cycles is a fundamental step in managing cyclical symptoms that affect quality of life, such as premenstrual syndrome, and in making informed reproductive health decisions [3,5]. However, historical and sociocultural barriers, together with inadequacies in reproductive health education, have been reported to contribute to insufficient knowledge and awareness among women of reproductive age worldwide regarding menstrual cycle physiology, ovulation timing, and fertility processes [6,7].

Traditionally, cycle tracking has been performed using pen-and-paper methods or has relied entirely on memory. These approaches may lead to data loss, retrospective recall bias, and substantial inaccuracies in estimating the fertile period [8]. Over the past decade, the global expansion of smartphone use and rapid developments in mobile health technologies have led to the emergence of a multibillion-dollar industry in women’s health technology, commonly referred to as “femtech” [9,10]. Menstrual cycle tracking applications are now among the most popular categories of mobile health applications and are actively used by hundreds of millions of women worldwide [3,7]. These applications enable women to digitally record cycle dates, monitor physical and psychological symptoms, receive personalised cycle analyses, and obtain predictions regarding future menstrual periods and peak fertility windows [8,11]. In the literature, these digital self-monitoring tools are regarded as important innovations with the potential to help women better understand their bodies, enhance their control over reproductive health, and support patient empowerment [11,12].

Evidence concerning the relationship between MCTA use and fertility knowledge is mixed. Application users have shown greater recognition of the fertile period and greater awareness of menstruation, premenstrual symptoms, and ovulation in observational studies [5,6]. Knowledge and attitudes also increased following application use in a quasi-experimental study [3], and fertility applications may support couples who are planning pregnancy [13]. Nevertheless, these findings do not establish that application use causes greater knowledge or awareness, because women with greater pre-existing interest or knowledge may be more likely to use these tools.

Concerns also remain regarding the scientific quality and transparency of commercial applications [9,10,11,14]. Many rely on calendar-based assumptions that do not account for individual variation in cycle length or ovulation timing [8,10,15]. Such predictions may be inaccurate when used for contraception or conception, whereas methods incorporating physiological indicators, including basal body temperature, cervical mucus, or luteinising hormone, may provide more reliable estimates [7,8,9,10,13].

Evidence remains limited among sociodemographically diverse women of reproductive age attending gynecology outpatient clinics. In particular, few studies in Türkiye have examined whether MCTA use, duration, frequency, and purpose are associated with fertility health knowledge and fertility awareness using validated measures [16,17]. Addressing this gap may clarify how different patterns of application engagement relate to these outcomes in a clinical population.

This study aimed to examine the associations of MCTA use, duration, frequency, and purpose with fertility health knowledge and fertility awareness among women of reproductive age attending a gynecology outpatient clinic. Specifically, the objectives were to: (1) determine the prevalence of MCTA use; (2) compare fertility health knowledge and fertility awareness between MCTA users and non-users; (3) examine the associations of duration and frequency of MCTA use with fertility health knowledge and fertility awareness; and (4) examine whether the purpose of MCTA use was associated with these outcomes. Data were collected using a literature-based Descriptive Information Form, together with the valid and reliable Fertility Health Knowledge Scale [16] and Fertility Awareness Scale [17]. The findings are expected to guide professionals working in women’s health nursing, midwifery, and primary healthcare services, while also contributing to the development of more reliable, evidence-based, and personalised digital health applications.

## 2. Materials and Methods

### 2.1. Study Design

This study aimed to examine the associations of MCTA use, duration, frequency, and purpose with fertility health knowledge and fertility awareness among women of reproductive age attending a gynecology outpatient clinic. The conduct and reporting of the study followed the Strengthening the Reporting of Observational Studies in Epidemiology (STROBE) guidelines [18].

### 2.2. Sample

The study population consisted of women aged 18–49 years who attended the gynecology outpatient clinic of a private hospital in Istanbul, Türkiye. During the data-collection period, women attending the gynecology outpatient clinic were informed about the study. Women who met the eligibility criteria and voluntarily agreed to participate were included using convenience sampling. Each participant was permitted to complete the questionnaire only once. Women aged between 18 and 49 years who were able to complete the data collection forms were eligible. Women who were receiving fertility treatment, had given birth within the last 6 months, or had a serious endocrinological or gynecological disease affecting the menstrual cycle, such as polycystic ovary syndrome, hypothyroidism, or hyperprolactinemia, were excluded from the study.

The minimum required sample size was calculated using G*Power version 3.1.9.7. Since the primary comparison of the study was based on menstrual cycle tracking application use status, the calculation was performed using the independent samples *t*-test. The analysis was conducted with a two-tailed test, a medium effect size (Cohen’s d = 0.50), an alpha error probability of 0.05, a power of 0.80, and an allocation ratio of 1:1 for estimation purposes. The minimum required sample size was calculated as 128 participants. Considering possible missing or incomplete data, this number was increased by 10%, resulting in a minimum target sample size of approximately 142 participants. Data collection continued throughout the planned study period, and all eligible women who agreed to participate were included. Thus, the study was completed with 484 women. The independent-samples *t*-test was selected because the prespecified primary analysis was the comparison of FHKS and FAS scores between MCTA users and non-users; correlation and regression analyses were secondary analyses. The final sample of 484 participants substantially exceeded the minimum required sample size. A sensitivity assessment indicated that, with *N* = 484, a two-sided alpha level of 0.05, and 80% power, the study was capable of detecting correlations of approximately r = 0.13 or greater, supporting the adequacy of the sample for the principal association analyses.

### 2.3. Data Collection

Data were collected between 1 February 2026 and 1 June 2026 using a face-to-face self-report questionnaire method. Participants who met the inclusion criteria were informed about the purpose and scope of the study. After providing informed consent, participants completed the questionnaire forms in the clinical setting. The data collection process took approximately 10–15 min for each participant. Questionnaires were completed individually in a private setting to protect confidentiality. The researcher provided clarification when needed but did not direct or influence participants’ responses.

Participants completed a Descriptive Information Form, the Fertility Awareness Scale, and the Fertility Health Knowledge Scale. The Descriptive Information Form included questions on sociodemographic characteristics, reproductive history, menstrual characteristics, contraceptive method use, menstrual cycle regularity, menstrual cycle tracking application use, duration and frequency of application use, and purpose of application use. Questions specific to menstrual cycle tracking application use were administered only to women who reported using such applications. Menstrual cycle tracking application use was assessed via a single self-report item asking whether participants were using a menstrual cycle tracking application. Participants who answered affirmatively were classified as MCTA users; those who answered negatively were classified as non-users.

### 2.4. Data Collection Instruments

Three instruments were used to collect data in this study: the Descriptive Information Form, the Fertility Awareness Scale, and the Fertility Health Knowledge Scale.

The Descriptive Information Form was developed by the researchers based on the literature [5,19,20]. The form included questions assessing participants’ sociodemographic, reproductive, menstrual, and menstrual cycle tracking application-related characteristics. These questions included age, education level, marital status, income status, pregnancy history, menstrual cycle regularity, contraceptive method use, menstrual cycle tracking application use, duration of application use, frequency of application use, and purpose of application use. Application-specific questions recorded the name of the application, duration of use (0–3 months, 4–6 months, 7–12 months, or ≥12 months), frequency of use (only during menstruation, several times per month, several times per week, or daily), the main information provided (menstrual-delay prediction, ovulation timing, PMS prediction, symptom analysis, or health advice), and the primary purpose of use (menstrual-date tracking, pregnancy planning/prevention, educational content, or symptom diary). Participants who reported that they had stopped using an application were classified as non-users. The form was used as a descriptive questionnaire rather than as a psychometric scale. No separate expert-panel review, formal content-validity assessment, cognitive interviewing, pilot testing, or reliability analysis was conducted for the researcher-developed items.

The Fertility Health Knowledge Scale was developed by Barron et al. [21] to evaluate fertility health knowledge and facilitate discussions about fertility health. The scale assesses biological knowledge of sex-specific reproductive health, including the menstrual cycle, sperm survival and quality, and erectile function, as well as knowledge of nonmodifiable and modifiable factors affecting fertility, such as age, body weight, stress, cannabis and tobacco use or exposure, caffeine intake, diet, exercise, and sleep habits. The scale consists of 30 items and has a single-factor structure. Items are rated on a five-point Likert-type scale ranging from 1 = definitely no to 5 = definitely yes. Responses of “definitely yes” and “probably yes” are considered correct. Item 20 is reverse-coded. The Turkish validity and reliability study of the scale was conducted by Sümen and Teskereci [16], and Cronbach’s alpha coefficient was reported as 0.949. In the present study, Cronbach’s alpha coefficient for the scale was 0.964. For calculation of the total score, the item responses were not dichotomized. Responses were scored from 1 to 5 as selected; therefore, incorrect and uncertain responses contributed their corresponding Likert scores. Item 20 was reverse-scored (1 = 5, 2 = 4, 3 = 3, 4 = 2, and 5 = 1), and all 30 item scores were summed. The possible total score ranges from 30 to 150, with higher scores consistently indicating greater fertility health knowledge.

The Fertility Awareness Scale was developed by Özşahin and Aksoy Derya [17] to assess fertility awareness among women. The scale is a 19-item Likert-type instrument consisting of two subdimensions: Body Awareness and Cognitive Awareness. The Body Awareness subdimension includes 10 items, and the Cognitive Awareness subdimension includes 9 items. Each item is scored from 1 to 5, with response options ranging from 1 = never to 5 = always. The scale has no reverse-coded items. The total score ranges from 19 to 95, with higher scores indicating higher fertility awareness. Scores between 19 and 43 indicate low awareness, scores between 44 and 69 indicate moderate awareness, and scores between 70 and 95 indicate high awareness. The possible score range is 10–50 for the Body Awareness subdimension and 9–45 for the Cognitive Awareness subdimension. In the original study, Cronbach’s alpha coefficients were reported as 0.887 for the total scale. In the present study, Cronbach’s alpha coefficient was 0.897 for the total scale. These cut-off categories were established in the original instrument-development study using the score-range average method [17]; they were not created by the authors of the present study.

### 2.5. Data Analysis

Data were analyzed using IBM SPSS Statistics version 25. The normality of continuous variables was evaluated by examining skewness and kurtosis values. Descriptive statistics were presented as numbers, percentages, means, and standard deviations. Independent samples *t*-tests were used to compare scale scores between two groups, and one-way analysis of variance was used for comparisons involving more than two groups. Tukey post hoc analysis was performed to identify the specific groups contributing to significant differences in ANOVA. Pearson correlation analysis was used to examine relationships between continuous variables. Simple linear regression analysis was performed to examine the association between menstrual cycle tracking application use and Fertility Health Knowledge Scale and Fertility Awareness Scale scores. Statistical significance was set at *p* < 0.05 for all analyses. Menstrual cycle tracking application use was entered into the regression models as a dichotomous variable (0 = user, 1 = non-user). Data were checked for completeness before analysis. No missing data were identified in the variables included in the analyses. Categorical characteristics were compared between the MCTA-use groups using Pearson’s chi-square test. Fisher’s exact test was used for education level after categories were combined because of sparse expected cell counts. Because the sole predictor in each model was dichotomous, the models estimated the mean difference between MCTA users and non-users rather than a continuous linear dose–response relationship. Each participant was permitted to complete the questionnaire only once, reducing the risk of duplicate observations; observations were treated as independent at the participant level.

### 2.6. Ethical Approval

Ethical approval was obtained from the Fenerbahçe University Ethics Committee (Approval No. 137.2025fbu; 23 December 2025). Institutional permission was obtained from the hospital where the study was conducted. Permission to use the study instruments was obtained from the respective scale developers. The study was conducted in accordance with the Declaration of Helsinki. All participants received information about the study purpose and procedures and provided written informed consent before data collection. Participation was voluntary, and participants were informed of their right to withdraw at any time without penalty.

## 3. Results

The participants in this study had a mean age of 28.55 ± 6.65 years (range: 18–44 years). Significant differences by education level were found for FHKS total scores (*p* = 0.021) and FAS cognitive awareness scores (*p* = 0.004). FAS cognitive awareness scores also differed significantly by marital status (*p* = 0.006) and contraceptive method use (*p* = 0.012). No significant differences were found in the scale scores according to income level, menstrual cycle regularity, or pregnancy status (all *p* > 0.05). Participants who used menstrual cycle tracking applications had significantly higher FHKS total, FAS cognitive awareness, FAS body awareness, and FAS total scores than non-users (all *p* < 0.001). Among MCTA users, FHKS total scores differed significantly according to the purpose of application use (*p* = 0.027), whereas the corresponding FAS comparisons were not statistically significant (all *p* > 0.05; Table 1). Additional comparisons showed no significant differences between the MCTA-use groups in education level (Fisher’s exact test, *p* = 0.343), marital status (χ^2^ = 0.030, *p* = 0.863), or menstrual regularity (χ^2^ = 0.009, *p* = 0.923). However, income level differed significantly between the groups (χ^2^ = 8.090, *p* = 0.018). Among 195 current MCTA users, the most frequently reported applications were Apple Health (*n* = 52, 26.7%), Flo (*n* = 32, 16.4%), calendar-based applications such as Takvimim/Takvim/My Calendar (*n* = 30, 15.4%), Pepapp (*n* = 24, 12.3%), Clue (*n* = 22, 11.3%), and P.C. (*n* = 20, 10.3%); other applications or note-taking tools accounted for 15 users (7.7%). Among 194 users with valid duration data, 34 (17.5%) had used an application for 0–3 months, 42 (21.6%) for 4–6 months, 24 (12.4%) for 7–12 months, and 94 (48.5%) for ≥12 months. Use frequency was reported as only during menstruation by 80 users (41.0%), several times per month by 45 (23.1%), several times per week by 33 (16.9%), and daily by 37 (19.0%). The main information provided was menstrual-delay prediction (*n* = 69, 35.6%), ovulation timing (*n* = 53, 27.3%), PMS prediction (*n* = 47, 24.2%), symptom analysis (*n* = 24, 12.4%), or health advice (*n* = 1, 0.5%) among 194 valid responses. The primary purpose was menstrual-date tracking (*n* = 150, 76.9%), pregnancy planning/prevention (*n* = 43, 22.1%), educational content (*n* = 1, 0.5%), or symptom diary use (*n* = 1, 0.5%).

Although statistically significant, the positive correlation between age and FAS total scores was small in magnitude (r = 0.150, *p* < 0.001). The positive correlation between menstrual cycle length and FHKS total scores was also statistically significant but small in magnitude (r = 0.113, *p* = 0.013), whereas no significant correlation was found between menstrual cycle length and FAS total scores (*p* > 0.05). Similarly, the statistically significant positive correlations of the duration of menstrual cycle tracking application use with FHKS total scores (r = 0.171, *p* < 0.001) and FAS total scores (r = 0.144, *p* = 0.001) were small in magnitude. The statistically significant positive correlations of the frequency of menstrual cycle tracking application use with FHKS total scores (r = 0.217, *p* < 0.001) and FAS total scores (r = 0.119, *p* = 0.009) were likewise small in magnitude. In addition, a moderate positive correlation was observed between FHKS total and FAS total scores (r = 0.411, *p* < 0.001; Table 2). A strong positive correlation was observed between duration and frequency of MCTA use (r = 0.727, *p* < 0.001), indicating that participants with longer durations of use also tended to report more frequent application use.

The findings of the simple regression analysis examining the association between menstrual cycle tracking application use and fertility health knowledge and fertility awareness are shown in Table 3. Menstrual cycle tracking application use was significantly associated with FHKS total scores and explained 8.5% of the variance in the regression model (*p* < 0.001, F = 44.771; B = −13.577, R^2^ = 0.085). Similarly, menstrual cycle tracking application use was significantly associated with FAS total scores and explained 6.6% of the variance in the regression model (*p* < 0.001, F = 33.884; B = −7.404, R^2^ = 0.066). These findings indicate that participants who used menstrual cycle tracking applications had higher fertility health knowledge and fertility awareness scores than non-users. Because users were coded as 0 and non-users as 1, the negative coefficients indicate that non-users had expected FHKS and FAS scores 13.577 and 7.404 points lower, respectively, than users.

## 4. Discussion

This study examined the associations of menstrual cycle tracking application (MCTA) use, duration, frequency, and purpose with fertility health knowledge and fertility awareness. MCTA users had higher Fertility Health Knowledge Scale (FHKS) and Fertility Awareness Scale (FAS) scores than non-users, and MCTA use accounted for 8.5% and 6.6% of the variance in these outcomes, respectively. These findings are consistent with studies reporting greater fertility knowledge or menstrual awareness among reproductive-health application users [3,5,6]. Digital tracking may encourage attention to cycle timing and bodily symptoms through personalized records and notifications [8,17]. However, the cross-sectional design does not establish that application use caused the observed differences. The negative direction of the regression coefficients reflects the coding scheme (0 = user, 1 = non-user) and therefore represents lower predicted scores among non-users, rather than a negative association of application use itself.

Purpose and intensity of use were also associated with the outcomes. Women using MCTAs as a symptom diary had higher FHKS scores than those using them only for ovulation tracking, whereas menstrual-cycle tracking users had intermediate scores. Recording physical and emotional symptoms may involve more active self-monitoring than using an application solely as a calendar [12,14]. Longer and more frequent use was positively, although weakly, correlated with both FHKS and FAS scores. FHKS and FAS scores were moderately correlated, suggesting that greater fertility knowledge co-occurred with greater awareness. Age was weakly associated with FAS scores, and menstrual bleeding duration was weakly associated with FHKS scores; these relationships may reflect accumulated reproductive experience or greater motivation to seek cycle-related information [11,12]. Although duration and frequency of MCTA use were strongly correlated, they represent distinct dimensions of application engagement: duration reflects how long the application had been used, whereas frequency reflects how often it was used. These variables were not entered simultaneously in a multivariable regression model.

Education level, marital status, and contraceptive use were also related to selected knowledge or awareness scores. University graduates had higher FHKS and cognitive-awareness scores, while married women and contraceptive users had higher cognitive-awareness scores. These differences may reflect variation in health literacy, access to information, and engagement in reproductive decision-making [6,9,11,12,14]. Nevertheless, education and marital status did not differ significantly between the MCTA-use groups. Income level did differ between groups and may have confounded the observed associations.

The findings support discussing digital cycle tracking during women’s health consultations, while recognizing that many commercial applications use opaque or insufficiently validated algorithms and standardized cycle assumptions [9,10]. Clinicians should explain that calendar-based fertility predictions may be inaccurate and should not be treated as definitive for contraception or conception. When appropriate, women may be directed toward evidence-based tools that transparently describe their methods and incorporate physiological indicators such as basal body temperature, cervical mucus, or hormone monitoring [3,8,9]. Symptom-recording and educational features may support self-monitoring, but their effectiveness requires further evaluation [3,14].

Strengths of this study include its sample of 484 reproductive-age women attending a gynecology outpatient clinic and the use of two validated scales [16,17,21]. Its limitations include convenience sampling at a single center, which restricts generalizability; the cross-sectional design, which precludes causal or directional inference; and self-reported data, which may be affected by recall or social-desirability bias. Women with greater pre-existing knowledge or awareness may have been more likely to use MCTAs. Although application names were recorded, application quality, technical algorithms, payment models, participants’ reliance on application predictions for reproductive decision-making, and the use of physiological fertility indicators such as basal body temperature, cervical mucus, or LH testing were not assessed [11]. Actual exposure to or engagement with educational content within the applications was not measured. In addition, the regression models were unadjusted, and residual confounding—particularly by income—cannot be excluded. The associations should therefore not be interpreted as independent effects. Furthermore, women attending a gynecology outpatient clinic may have had greater reproductive health concerns, previous gynecological experiences, or motivation to seek reproductive health information than women in the general population. This may have introduced selection bias and further limits the generalizability of the findings. In addition, the researcher-developed Descriptive Information Form did not undergo formal content-validity or reliability testing; therefore, measurement error in the MCTA-related questions cannot be excluded. Observations were treated as independent at the participant level; however, separate diagnostic outputs for homoscedasticity, residual normality, and influential observations were not available for the present revision. Therefore, the regression findings should be interpreted with this limitation in mind.

## 5. Conclusions

This study showed that menstrual cycle tracking application (MCTA) use was significantly associated with higher fertility health knowledge and fertility awareness among women of reproductive age attending a gynecology outpatient clinic (18–49 years, *N* = 484). In the regression models, MCTA use explained 8.5% of the variance in fertility health knowledge (R^2^ = 0.085) and 6.6% of the variance in fertility awareness (R^2^ = 0.066; *p* < 0.001). Women who used MCTAs had significantly higher fertility knowledge and awareness scores than non-users (*p* < 0.001). Longer duration and greater frequency of MCTA use were positively correlated with fertility health knowledge and fertility awareness. Women who used the application as a “Symptom Diary,” regularly recording physical and emotional symptoms, had the highest fertility health knowledge scores (117.35 ± 18.78).

In conclusion, menstrual cycle tracking applications may support self-monitoring and reproductive health education, but they should not be recommended uncritically. Commercial applications vary in scientific validity and algorithmic transparency, and calendar-based predictions should not be used as the sole basis for contraceptive or conception decisions. Women’s health professionals may discuss application use and, where appropriate, guide women toward evidence-based tools that transparently describe their methods, limitations, and use of physiological fertility indicators. Longitudinal and experimental studies are needed to determine whether MCTA use leads to improvements in fertility knowledge or awareness.

## Figures and Tables

**Table 1:** Fertility awareness and knowledge scores according to sociodemographic, menstrual, and reproductive characteristics.

	FHKS	FAS		
	Total	Cognitive Awareness	Body Awareness	Total
	Mean ± SD	Mean ± SD	Mean ± SD	Mean ± SD
**Education level**				
Literate (*n* = 5, 1.0%)	105.80 ± 24.67	23.00 ± 7.52	29.80 ± 12.97	52.80 ± 19.89
Secondary education (*n* = 61, 12.6%)	110.76 ± 24.07	27.11 ± 7.59	34.35 ± 8.85	61.46 ± 15.22
University graduate (*n* = 418, 86.4%)	105.01 ± 20.83	29.02 ± 6.44	34.25 ± 7.25	63.28 ± 12.56
F/p	3.879/**0.021**	5.648/**0.004**	0.760/0.468	2.125/0.121
**Marital status**				
Married (*n* = 161, 33.3%)	105.60 ± 20.13	29.23 ± 6.64	33.61 ± 7.58	62.84 ± 13.31
Single (*n* = 323, 66.7%)	109.20 ± 23.88	27.34 ± 7.30	34.58 ± 8.45	61.92 ± 14.50
t/p	−1.644/0.101	2.763/**0.006**	−1.234/0.218	0.672/0.502
**Income level**				
Income less than expenses (*n* = 133, 27.5%)	107.22 ± 22.58	27.55 ± 6.95	34.31 ± 8.41	61.86 ± 14.32
Income equal to expenses (*n* = 299, 61.8%)	108.56 ± 22.88	28.22 ± 7.18	34.47 ± 8.01	62.69 ± 13.86
Income greater than expenses (*n* = 52, 10.7%)	106.79 ± 22.66	27.58 ± 7.40	32.92 ± 8.58	60.50 ± 15.09
F/p	0.242/0.785	0.499/0.608	0.793/0.453	0.597/0.551
**Menstrual regularity**				
Regular (*n* = 375, 77.5%)	107.78 ± 22.44	28.25 ± 7.02	34.41 ± 8.01	62.66 ± 13.84
Irregular (*n* = 109, 22.5%)	108.77 ± 23.85	26.99 ± 7.48	33.75 ± 8.76	60.74 ± 14.99
t/p	−0.402/0.688	1.628/0.104	0.733/0.464	1.248/0.213
**Contraceptive method use**				
Yes (*n* = 138, 28.5%)	108.15 ± 20.99	29.26 ± 6.90	34.33 ± 7.90	63.59 ± 13.70
No (*n* = 346, 71.5%)	107.94 ± 23.44	27.45 ± 7.17	34.22 ± 8.30	61.68 ± 14.25
t/p	0.093/0.926	2.529/0.012	0.127/0.899	1.347/0.179
**Pregnancy status**				
Yes (*n* = 108, 22.3%)	109.31 ± 20.88	28.81 ± 7.96	34.60 ± 9.15	63.40 ± 15.99
No (*n* = 376, 77.7%)	107.52 ± 22.13	30.11 ± 6.55	34.46 ± 6.34	64.57 ± 12.01
t/p	0.428/0.670	−0.922/0.358	0.087/0.931	−0.427/0.670
**Menstrual cycle tracking application use**				
Yes (*n* = 195, 40.3%)	113.30 ± 23.16	29.11 ± 8.05	36.01 ± 8.72	65.12 ± 15.46
No (*n* = 289, 59.7%)	99.72 ± 19.41	26.20 ± 4.93	31.52 ± 6.38	57.71 ± 10.21
t/p	6.691/**<0.001**	4.460/**<0.001**	6.117/**<0.001**	5.821/**<0.001**
**Purpose of menstrual cycle tracking application use**				
To track the menstrual cycle (*n* = 150, 76.9%)	113.52 ± 20.58 ^ab^	28.96 ± 7.58	35.20 ± 8.99	64.16 ± 15.49
Ovulation tracking (*n* = 43, 22.1%)	108.13 ± 29.54 ^a^	27.91 ± 8.80	35.42 ± 10.37	63.33 ± 15.49
Symptom diary (*n* = 2, 1.0%)	117.35 ± 18.78 ^b^	30.41 ± 7.84	37.44 ± 6.57	67.85 ± 12.54
F/p	3.662/**0.027**	2.250/0.107	1.984/0.139	2.312/0.101

**Note**: Bold values indicate statistical significance (*p* < 0.05). Superscript letters indicate Tukey post hoc comparisons. t/p indicates the independent-samples *t*-test statistic and its corresponding *p*-value; F/p indicates the one-way analysis of variance (ANOVA) F statistic and its corresponding *p*-value. **Abbreviations**: FHKS, Fertility Health Knowledge Scale; FAS, Fertility Awareness Scale; SD, standard deviation.

**Table 2:** Correlations between selected continuous variables and FHKS and FAS scores.

Variables	Mean ± SD	r/p ^a^	Age	Menstrual Bleeding Duration (Days)	Duration of MCTA Use	Frequency of MCTA Use	FHKS Total	FAS Total
**Age**	28.55 ± 6.65	r	1	−0.320	0.008	0.030	−0.037	0.150
		p		**<0.001**	0.867	0.506	0.414	**<0.001**
**Menstrual bleeding duration (days)**	5.55 ± 1.65	r	−0.320	1	−0.013	−0.026	0.113	−0.074
		p	**<0.001**		0.778	0.574	**0.013**	0.102
Duration of MCTA use (ordinal category score; users only, *n* = 194)	2.83 ± 1.74	r	0.008	−0.013	1	0.727	0.171	0.144
		p	0.867	0.778		**<0.001**	**<0.001**	**0.001**
Frequency of MCTA use (ordinal category score; users only, *n* = 195)	2.80 ± 1.69	r	0.030	−0.026	0.727	1	0.217	0.119
		p	0.506	0.574	**<0.001**		**<0.001**	**0.009**
**FHKS**	108.00 ± 22.74	r	−0.037	0.113	0.171	0.217	1	0.411
		p	0.414	**0.013**	**<0.001**	**<0.001**		**<0.001**
**FAS**	62.23 ± 14.11	r	0.150	−0.074	0.144	0.119	0.411	1
		p	**<0.001**	0.102	**0.001**	**0.009**	**<0.001**	

**Note**: Bold values indicate statistical significance (*p* < 0.05). ᵃ Pearson correlation test. Among MCTA users, duration of application use was coded as an ordinal category score (1 = 0–3 months, 2 = 4–6 months, 3 = 7–12 months, and 4 = ≥12 months), and frequency of application use was coded as an ordinal category score (1 = only during menstruation, 2 = several times per month, 3 = several times per week, and 4 = daily). Age, menstrual bleeding duration, FHKS, and FAS were measured in all participants (*n* = 484). Duration of MCTA use was assessed among 194 users with valid duration data, whereas frequency of MCTA use was assessed among 195 users; correlations involving each variable were calculated using its corresponding user subgroup. **Abbreviations**: FHKS, Fertility Health Knowledge Scale; FAS, Fertility Awareness Scale; SD, standard deviation; r, correlation coefficient.

**Table 3:** Simple linear regression analysis of the association between menstrual cycle tracking application use and FHKS and FAS scores.

Dependent Variables	Independent Variables	Unstandardized Coefficients		Standardized Coefficients	t	*p*	VIF
		B (95% CI)	Standard Error	β			
FHKS Total	Using a menstrual cycle tracking application	−13.577 [−17.56, −9.59]	2.029	−0.292	−6.691	**<0.001**	1.000
FAS Total		−7.404 [−9.90, −4.91]	1.272	−0.256	−5.821	**<0.001**	1.000

**Note**: For FHKS Total, ANOVA test regression (F) = 44.771; *p* < 0.001; R^2^ = 0.085; adjusted R^2^ = 0.083. For FAS Total, ANOVA test regression (F) = 33.884; *p* < 0.001; R^2^ = 0.066; adjusted R^2^ = 0.064. Bold values indicate statistical significance (*p* < 0.05). **Abbreviations**: FHKS, Fertility Health Knowledge Scale; FAS, Fertility Awareness Scale; B, unstandardized regression coefficient; SE, standard error; β, standardized regression coefficient; VIF, variance inflation factor. CI, confidence interval; MCTA use was coded as 0 = user and 1 = non-user; therefore, users constituted the reference category.

## Data Availability

The data presented in this study are not publicly available due to ethical and privacy restrictions. De-identified data may be made available by the corresponding author upon reasonable request, subject to applicable ethical requirements.

## Abbreviations

The following abbreviations are used in this manuscript:

ANOVA	Analysis of Variance
FAS	Fertility Awareness Scale
FHKS	Fertility Health Knowledge Scale
MCTA	Menstrual Cycle Tracking Application
SD	Standard Deviation
SE	Standard Error
SPSS	Statistical Package for the Social Sciences
STROBE	Strengthening the Reporting of Observational Studies in Epidemiology
VIF	Variance Inflation Factor
